# A Practical Treatise on Diseases of Women

**Published:** 1892-04

**Authors:** 


### Book Reviews.

A PRACTICAL TREATISE ON DISEASES OF WOMEN.—By T. Gaillard Thomas, M. D., L. L. D., and Paul F. Munde, M. D. Published by Lea Brothers & Company, Philadelphia.

This work, formerly edited by Dr. Thomas, has been revised and brought up to date by Dr. Munde, incorporating some of his individual opinions, developed from a ripe clinical experience, thereby adding to the value of the book.

The reviser has retained the original peculiar manner of the author, in writing the work, which renders the subjects treated of more easily understood and comprehended by the student.

It presents a conservative view of pelvic inflammations, with the latest methods of treatment, and advises the saline method of after treatment of laparotomy, in preference to the opium plan of treatment.

The plate portraying the normal topography of the female genital organs is an advance over similar works, and comes nearer portraying the normal relation of the parts.

The flap splitting method of operating on the perineum is well presented, while the Emmet operation is not so well presented, including plate XC ; page 205.

The work certainly will be a conservative guide to the student, a good counsellor to the practitioner and of benefit to the specialist. R. R. K.

